# Immersive virtual reality for acupuncture knowledge and practical skills: a comparative educational study

**DOI:** 10.3389/fpubh.2026.1922688

**Published:** 2026-08-27

**Authors:** Lingyi Wu, Vigneshkumar Chellappa, Qi Xie, Xuan Mao, Yan Luximon, Riji Yu

**Affiliations:** 1 School of Art and Design, Hubei University, Wuhan, China; 2 School of Design, The Hong Kong Polytechnic University, Kowloon, Hong Kong SAR, China

**Keywords:** acupuncture, Chinese medicine, control group, healthcare education, virtual reality

## Abstract

**Background:**

Acupuncture education requires mastery of complex theoretical concepts and psychomotor skills, yet conventional teaching often struggles to bridge the gap between abstract knowledge and hands-on application. This study evaluated the association of an immersive virtual reality-based acupuncture training system, PainEaseVR, with posttest learning outcomes when used as a supplement to conventional acupuncture education.

**Methods:**

A stratified, randomized, posttest comparison study was conducted with 60 third-year undergraduate students majoring in Traditional Chinese Medicine. Participants were assigned to either an experimental group using PainEaseVR (*n* = 30) or a control group receiving conventional instruction (*n* = 30). Both groups completed 12 h of instruction, including 6 h of theoretical teaching and 6 h of practical training. Learning outcomes were assessed using a 12-item theoretical knowledge test, an 18-indicator practical skills assessment, and an 11-item student feedback questionnaire.

**Results:**

The VR group achieved higher scores in theoretical knowledge (M = 10.08, SD = 0.99) than the traditional group (M = 9.04, SD = 1.31). The VR group also outperformed the traditional group in practical skills (M = 32.52, SD = 2.28 vs. M = 28.62, SD = 2.77). Students in the VR group reported higher satisfaction, engagement, and confidence across all feedback items.

**Conclusion:**

PainEaseVR appears to be a promising supplementary tool for acupuncture education. The findings suggest that VR was associated with higher scores in acupuncture knowledge and practical skills, as well as a more engaging learning experience, within the context of this posttest comparison study.

## 1 Introduction

Acupuncture is a vital component of traditional Chinese medicine, having been extensively practiced in China for over three millennia (1). Today, acupuncture is widely recognized around the globe and is particularly effective for treating various chronic ailments, especially those associated with pain. This therapy utilizes fine needles to penetrate specific meridians, restoring the flow and balance of Qi and blood by stimulating designated acupuncture points. In Western countries, acupuncture has emerged as a compelling alternative treatment for chronic conditions such as asthma, migraines, stroke complications, menstrual disorders, knee and back pain, chemotherapy-induced nausea and vomiting, and hot flashes (1). Many individuals with chronic conditions turn to acupuncture when conventional treatments fail to provide relief (2). As such, adequate education and training in acupuncture are essential for practitioners (3). The number of conventional physicians pursuing acupuncture training has been steadily increasing (4), highlighting the need for such education to achieve optimal treatment outcomes, ensure patient safety, and uphold professional standards in the field.

Traditionally, acupuncture education and training have combined theoretical classroom instruction with supervised clinical practice. In the classroom, students learn about the historical roots of acupuncture, the physiological principles underpinning its benefits, and the intricate mapping of meridians and acupoints (5). This theoretical foundation is reinforced through practical instruction, in which students practice identifying acupoints and performing needling techniques under the guidance of experienced instructors. While this approach has produced competent practitioners, it is not without limitations. Access to training opportunities is often hindered by cost, safety concerns, and logistical challenges (3). Additionally, students frequently struggle to visualize complex anatomical features, particularly when reconciling two-dimensional textbook illustrations with three-dimensional human anatomy. This difficulty can impede their ability to accurately identify acupoints and understand the spatial relationships among various anatomical landmarks (6). Furthermore, translating theoretical knowledge into practical skills requires continuous practice and timely feedback, which may not always be feasible in traditional educational settings due to limited clinical placements, variability in patient presentations, and instructor availability. These issues may lead to inconsistencies in training quality, ultimately affecting students’ confidence and competence as they transition into professional practice.

In response to these challenges, educational institutions are increasingly exploring innovative pedagogical approaches, including integrating digital technologies to enhance learning outcomes (7). Among these technologies, virtual reality (VR) has emerged as a promising tool. VR provides immersive, interactive educational experiences that can replicate real clinical situations, allowing students to practice acupuncture procedures in a safe, controlled environment (8). This technology enables trainees to visualize anatomical structures in three dimensions, receive real-time feedback on their performance, and engage in repetitive practice without the risks associated with live patient interactions (9). By connecting theoretical knowledge with practical application, VR offers students the opportunity to develop both cognitive and psychomotor skills in an engaging and effective way.

Despite the growing interest in VR as an educational tool in healthcare, its application in acupuncture education and training remains comparatively underexplored. Most existing research on VR in medical education has focused on fields such as surgery, anatomy, and emergency care, demonstrating improvements in knowledge retention, skill acquisition, and learner engagement (10–14). However, there is limited research investigating the specific impact of VR on acupuncture education, particularly regarding its effectiveness in enhancing both theoretical understanding and practical skills (15). This gap is noteworthy, as acupuncture requires a unique combination of cognitive and psychomotor skills that could benefit from the immersive, repetitive practice environments VR provides. The present study extends this emerging body of research by evaluating an immersive VR platform that integrates theoretical instruction, acupoint learning, procedural rehearsal, and case-based diagnostic simulation into a single educational system. Accordingly, the aim of this study was to assess whether immersive VR-based acupuncture education was associated with higher posttest scores in students’ theoretical understanding and practical skills than a conventional teaching approach. The study was guided by two hypotheses.

*H1: Students receiving immersive VR-based training were expected to obtain significantly higher total scores on the 12-item theoretical knowledge test than students receiving conventional instruction.*

*H2: Students receiving immersive VR-based training were expected to obtain significantly higher total scores on the 18-item practical-skills assessment than students receiving conventional instruction.*

## 2 Literature review

### 2.1 Traditional acupuncture education and training

Acupuncture is a medical art, and achieving proficiency requires comprehensive training in operational skills (16). Conventional acupuncture education typically combines theoretical instruction and hands-on practice, with instructors serving as the primary authority for imparting knowledge. This traditional approach often relies on language and imagery to convey concepts, which can limit students’ engagement and enthusiasm, leading to a more abstract understanding of the material (17). Classroom interactions are often limited, making it challenging to foster students’ interest and motivation to learn. In addition to theoretical knowledge, medical professionals must be adept at executing needling techniques with the appropriate depth, angle, and intensity. Acupuncture manipulation encompasses the assessment and application of these parameters at designated acupoints. However, even when targeting the same acupoint, variations in technique can significantly affect outcomes, including nerve excitability, local oxygen tension, and chemical concentrations (18). These variations are often influenced by the practitioner’s experience, complicating the standardization of teaching methods and the effective acquisition of skills (3). Moreover, novices frequently experience apprehension when performing acupuncture procedures on human subjects (19), hindering their learning and confidence.

### 2.2 VR in acupuncture and healthcare education

The integration of VR technology has presented a promising solution for enhancing acupuncture training in recent decades. VR provides a realistic and immersive environment that enables practitioners to refine acupuncture skills. Specifically, VR can complement traditional training by enabling three-dimensional anatomical visualization, allowing repeated practice in diverse clinical scenarios, and imposing safety constraints during complex procedures, thereby reducing risk to patients and peers (15, 20). Previous research demonstrates that integrating VR into educational settings enhances learner engagement and motivation (21) and increases students’ enjoyment and self-efficacy (3, 22). VR supports a constructivist learning model by enabling learners to actively construct knowledge through integrating new information with prior experience and engaging in authentic, problem-based activities, rather than passively receiving information (23). Interactive simulations provide opportunities for learners to explore clinical scenarios, make decisions, observe outcomes, and refine their understanding through repeated practice and reflection (24), which enhances the overall training experience (25). The immersive nature of VR allows students to explore, experiment, and receive immediate feedback, enhancing their learning outcomes (26). Recent developments in consumer hardware and mobile technologies have broadened access to VR and related digital content through increasingly available consumer devices (27). However, the implementation of VR in educational settings still depends on hardware availability, interface design, institutional resources, and individual user requirements.

Acupuncture education encompasses critical knowledge of acupuncture points, manipulation techniques, and therapeutic scenarios, including meridian pathways and appropriate insertion angles (28). While traditional training has limitations as discussed above, VR can visually represent three-dimensional anatomy, including muscles, bones, blood vessels, and nerves (29). Skills acquired through various surgical simulation models are increasingly recognized for their effective transfer to clinical practice (19). For instance, Gao et al. (30) utilized the HTC VIVE VR device to develop a model for storing acupuncture point information, facilitating human-computer interaction and retrieval of relevant data. Lee et al. (19) examined the effectiveness of newly created phantom acupoint instruments for novice acupuncture students to practice manipulation techniques and enhance their skills. Sun et al. (31) discussed the application of mixed reality to improve acupuncture practice by implementing an acupuncture training simulator. However, to the author’s knowledge, no research has yet explored the use of VR to comprehensively cover the three fundamental aspects of acupuncture: acupuncture point information, manipulation techniques, and therapeutic scenarios.

## 3 PainEase VR

PainEaseVR is an immersive virtual acupuncture simulation system designed to provide safe, controllable, and repeatable educational experiences. It allows trainees to conduct acupuncture diagnostics and deepen their understanding of acupuncture principles and effects in a secure virtual environment. The system includes a comprehensive explanation of acupuncture, details on acupoints, manipulation techniques, therapeutic cases and scenarios, and a simulated examination platform to enhance learning of acupuncture procedures. It provides a platform for academics to examine the potential educational role of VR acupuncture in addressing specific conditions and improving training outcomes.

### 3.1 System’s navigation interface

**Figure 1:**
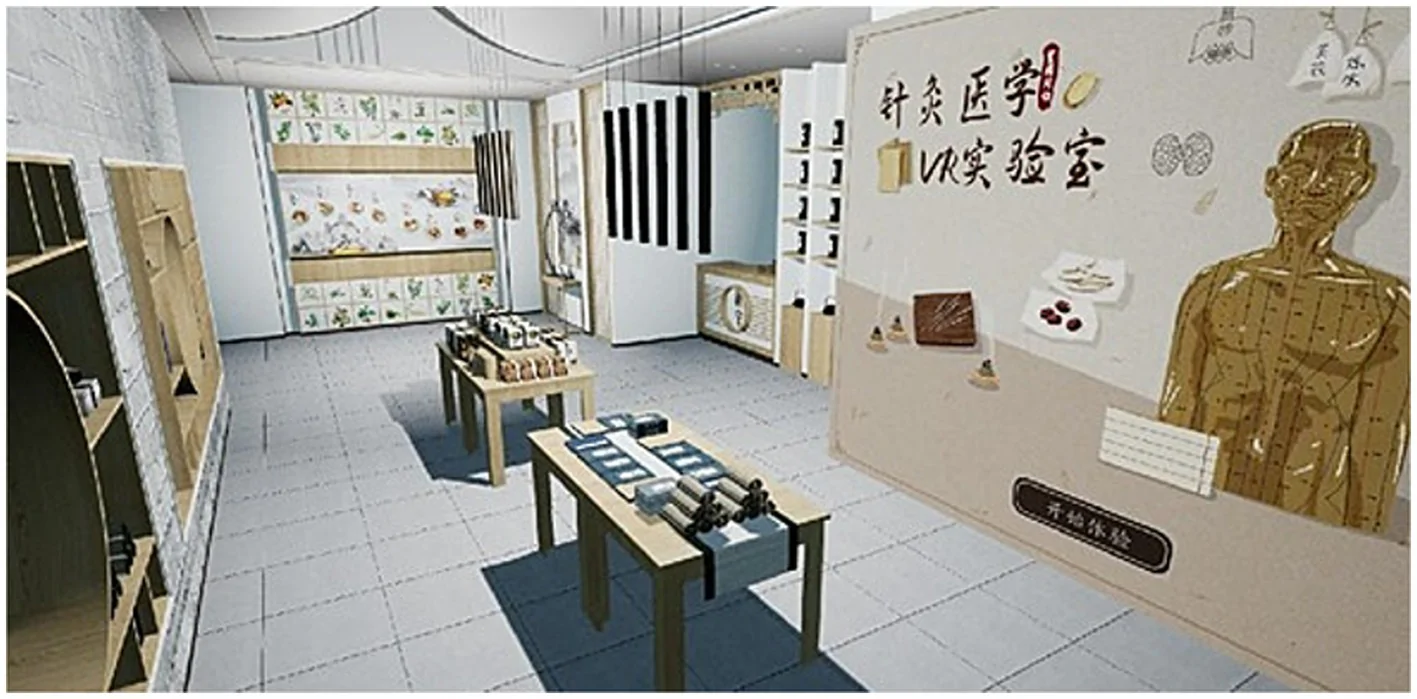
Main navigation interface of PainEaseVR, showing the central hub for selecting instructional, procedural-training, and diagnostic modules.

The navigation interface provides a comprehensive overview of the acupuncture training system. Designed for individuals of various educational backgrounds and age groups, it was intended to support accessibility and user-friendliness. The interface offers an immersive learning experience that enhances engagement and effectiveness. Users can navigate the system using a controller, exploring four interactive learning areas, each focused on different aspects of acupuncture practice and philosophy. These interactive settings enable users to engage actively with the material, thereby deepening their understanding of the subject. The system employs various methods to connect these learning spaces, creating a cohesive instructional experience (see Figure 1). A key feature is the voice prompts that guide users throughout the learning process, helping them understand specific acupuncture concepts in various contexts. These prompts not only provide timely and relevant information but also enhance user engagement, making the educational experience more enjoyable. This flexibility enables users to tailor their learning to their individual needs, thereby significantly improving their motivation. The approach promotes iterative learning, which is essential for consolidating knowledge and enhancing the practical application of acupuncture techniques. Repetition helps users reinforce their comprehension and build confidence in executing these techniques effectively.

### 3.2 Instructional scenario—virtual acupuncture training environment

**Figure 2:**
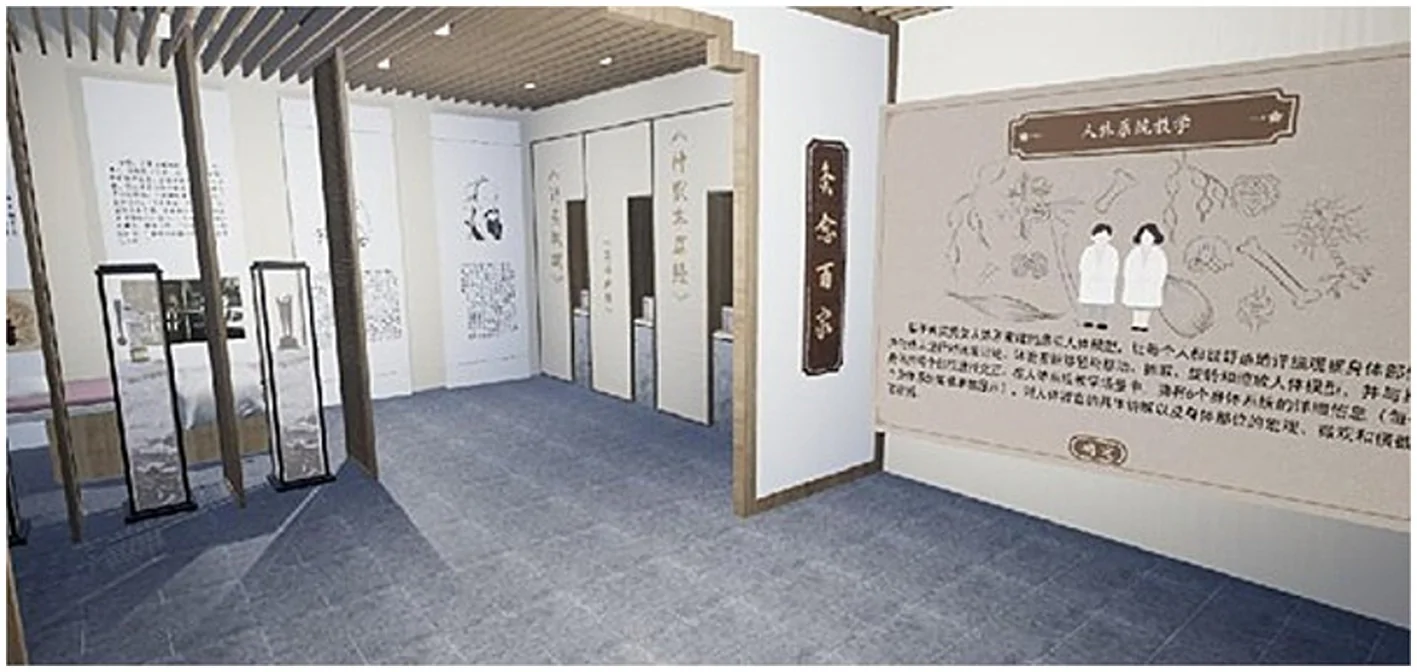
Instructional environment in PainEaseVR for teaching human body systems and historical background relevant to acupuncture.

The virtual acupuncture training environment is designed with a modern-retro acupuncture esthetic, featuring a compact, square room layout that blends contemporary design with historical elements (see Figure 2). This unique architectural style creates an immersive atmosphere, enhancing the educational experience by providing a location that is both visually appealing and intellectually stimulating. The room’s walls display historical depictions of ancient acupuncture practices, showcasing the evolution of this traditional medicinal technique. These visual elements include intricate images and descriptions of historical acupuncture methods, as well as portraits and biographies of influential figures who have shaped the field. By integrating these historical components into the design, the space serves as a living museum, offering users a deeper understanding of acupuncture’s rich legacy and cultural significance.

### 3.3 Instructional scenario—acupuncture learning module

**Figure 3:**
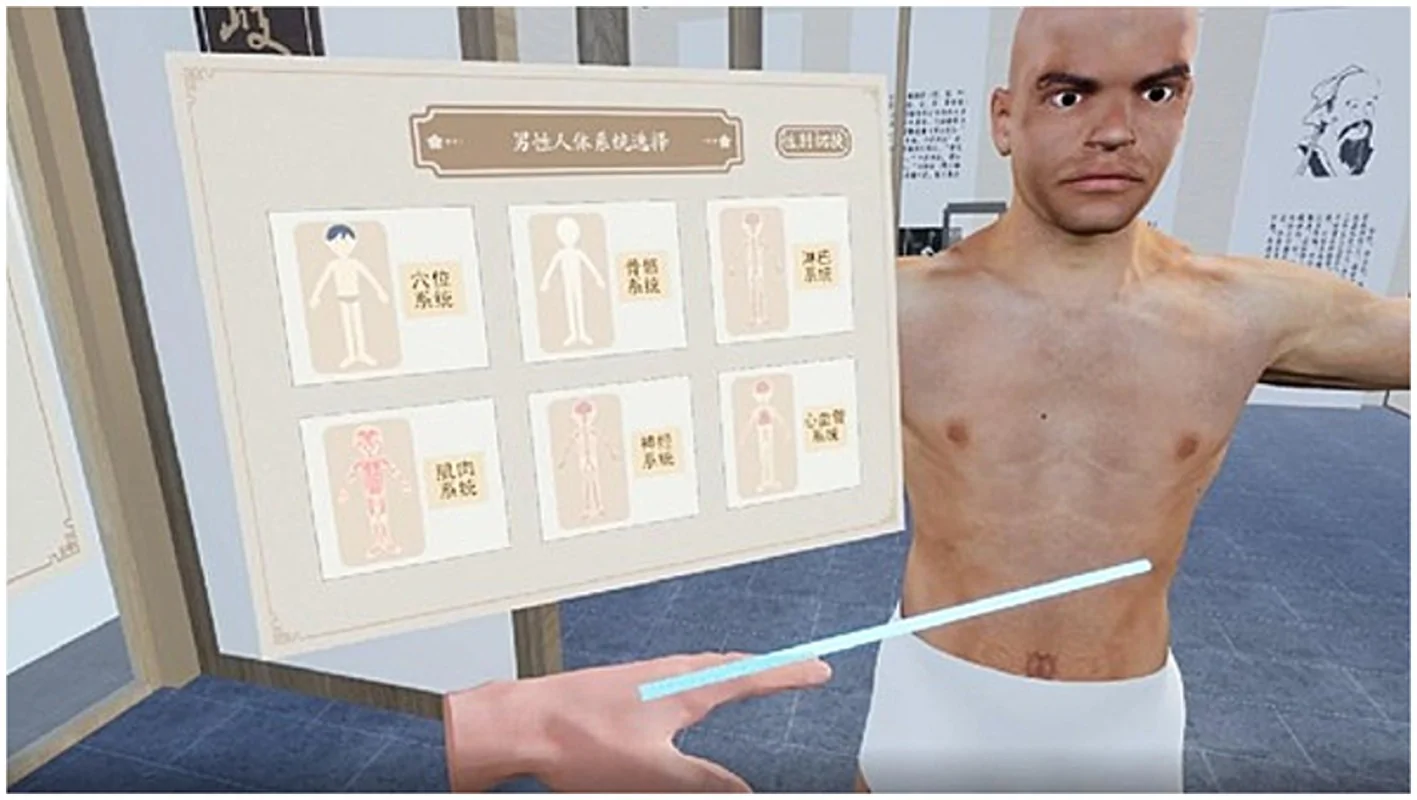
Acupoint teaching environment in PainEaseVR, illustrating interactive visualization of acupoint locations and associated explanatory information.

An acupuncture learning module provides a comprehensive overview of commonly used acupoints on the human body, helping users gain an in-depth understanding of these essential elements of acupuncture practice. Acupoints, also known as acupuncture points, are specific locations targeted during treatments to promote healing and balance within the body. The acupuncture learning module educates users about the significance and application of these points in traditional Chinese medicine. To enhance the learning experience, the module features an interactive touch interface that allows users to engage directly with the content. This interface combines user-friendly graphics and text to create an immersive educational tool. The graphics visually represent the acupoints, showing their precise locations on the body and their connections to various physiological and energetic pathways (see Figure 3). The VR system included custom three-dimensional human-body models for both male and female subjects. Relevant acupoints were manually annotated on the surface of each model rather than being automatically generated or approximated from an existing anatomical template. Each acupoint was implemented as an independent interactive object with a unique identifier and three-dimensional coordinates relative to the body model. To support efficient interaction during virtual needle insertion, the body model was spatially partitioned using an octree-based collision-detection structure. Accompanying text provides detailed explanations of each acupoint’s function, therapeutic benefits, and historical context, ensuring a well-rounded understanding. This interactive approach encourages users to explore theoretical concepts related to acupoints in an engaging and accessible manner. The touch interface promotes active participation, enabling users to navigate the information at their own pace and explore areas of interest in greater depth. This hands-on learning method enhances comprehension and aids retention, as users can visualize and interact with the content in a meaningful way.

### 3.4 Training scenario—acupuncture practice operation

**Figure 4:**
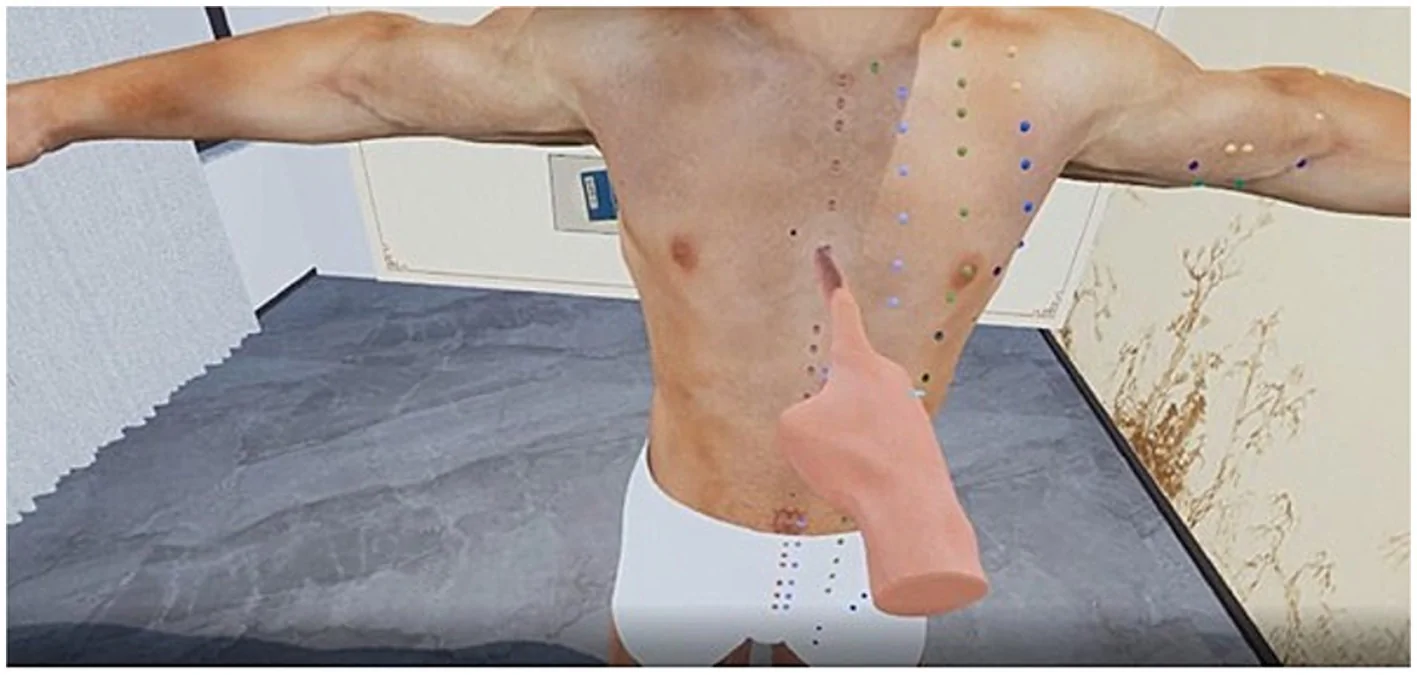
Example of finger-based interaction with an acupuncture point in PainEaseVR.

**Figure 5:**
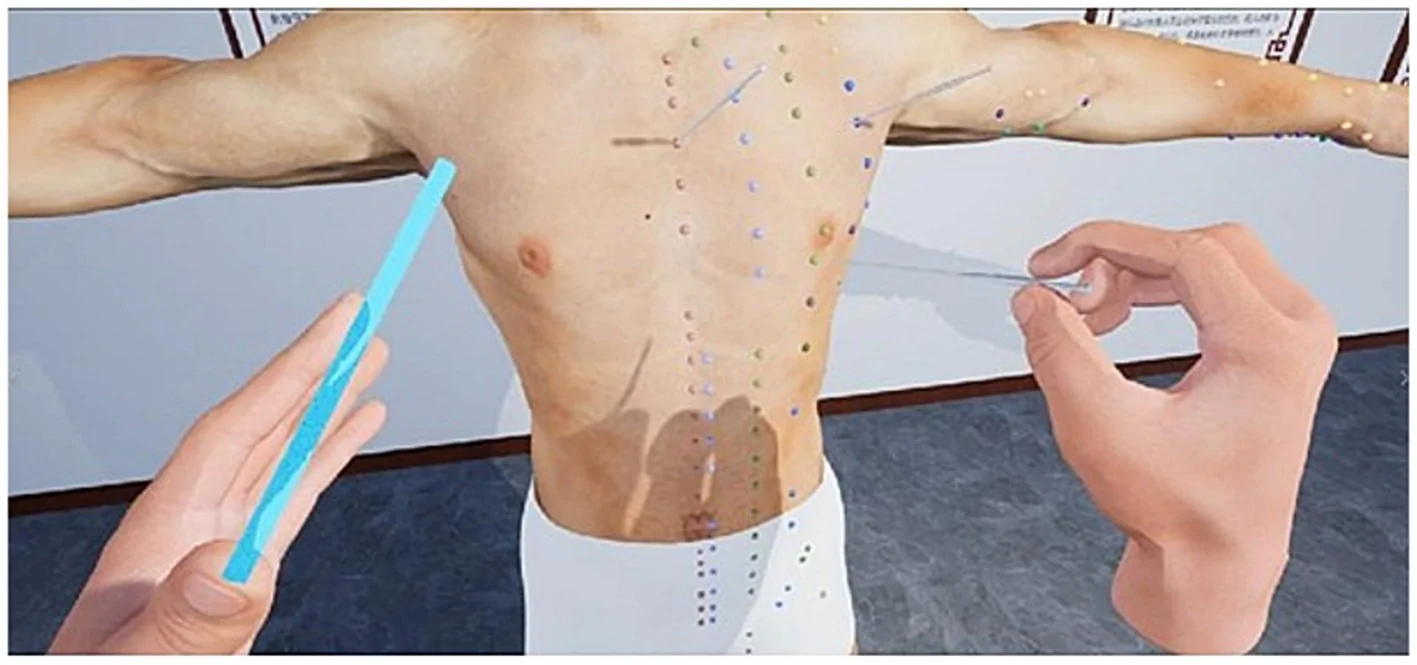
Practical training environment in PainEaseVR for simulated acupuncture operation.

**Figure 6:**
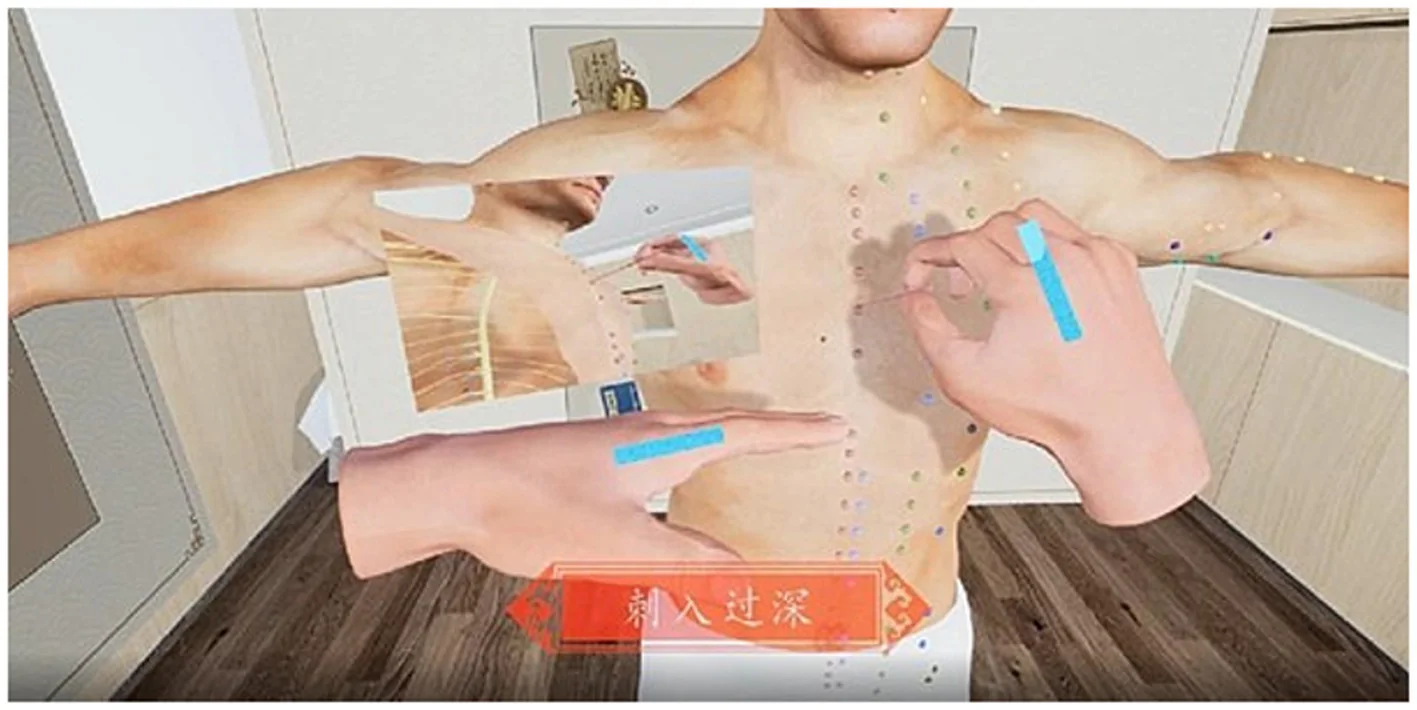
Hybrid collision-detection mechanism used in PainEaseVR to support interaction during acupuncture-skills training.

This innovative environment combines ancient acupuncture principles with advanced technology to enhance user engagement in the learning process (see Figures 4–6). This unique blend enables users to explore the rich traditions of acupuncture while benefiting from modern educational tools, creating an atmosphere that is both engaging and enlightening. Users will gain insights into the history, purpose, and application of these needles through detailed explanations and demonstrations, deepening their understanding of acupuncture. The environment emphasizes the importance of practical application and skill development alongside academic learning. Users are encouraged to practice continuously to refine their acupuncture skills in a supportive, interactive setting. This hands-on approach ensures that trainees can apply their theoretical knowledge in practical situations, fostering confidence and proficiency in their acupuncture practice.

### 3.5 Application scenario—VR-based needling training

In a practical needling scenario, the VR system initially presents the types, fundamental functions, and standardized handling methods of acupuncture needles. Subsequently, the learner selects a target acupoint and performs the needling procedure using the VR controller. The system continuously tracks the position and orientation of the virtual needle and employs a hybrid collision-detection method to determine the spatial relationship among the needle tip, the human model, and the target acupoint. The acupuncture needle is modeled using an Oriented Bounding Box (OBB)-based hierarchical bounding structure, while the human model and its acupoints are organized through four-level octree spatial partitioning. The octree structure is initially used to exclude spatial regions that cannot interact with the needle. Ray-based intersection detection is then applied to candidate acupoints to determine whether the needle tip has reached the intended target. Upon valid contact, the system generates haptic feedback through the controller and displays a magnified frontal view of the needling area near the learner’s left-hand controller, facilitating closer observation of the insertion position and direction. The system continuously monitors insertion depth in real time. Based on predefined depth thresholds for the selected acupoint, the interface provides prompts indicating whether the insertion is too shallow, within the appropriate range, or too deep. Performance is evaluated automatically according to predefined rules based on target-point accuracy and insertion depth relative to the acceptable range for the selected acupoint. This visual feedback, combined with the close-up view and haptic response, enables immediate adjustment of the needling operation.

### 3.6 Case bank diagnostic training scenario

**Figure 7:**
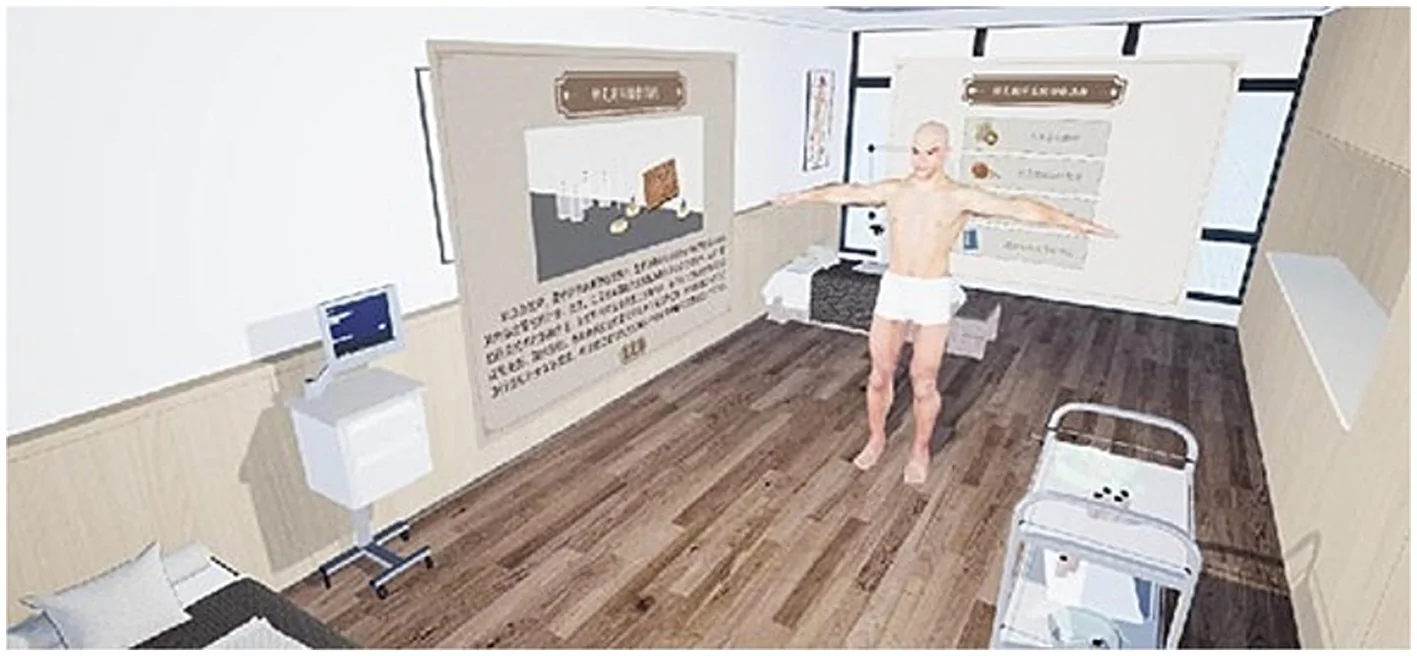
Case bank diagnostic-training environment in PainEaseVR, designed to simulate clinical assessment and decision-making tasks.

**Figure 8:**
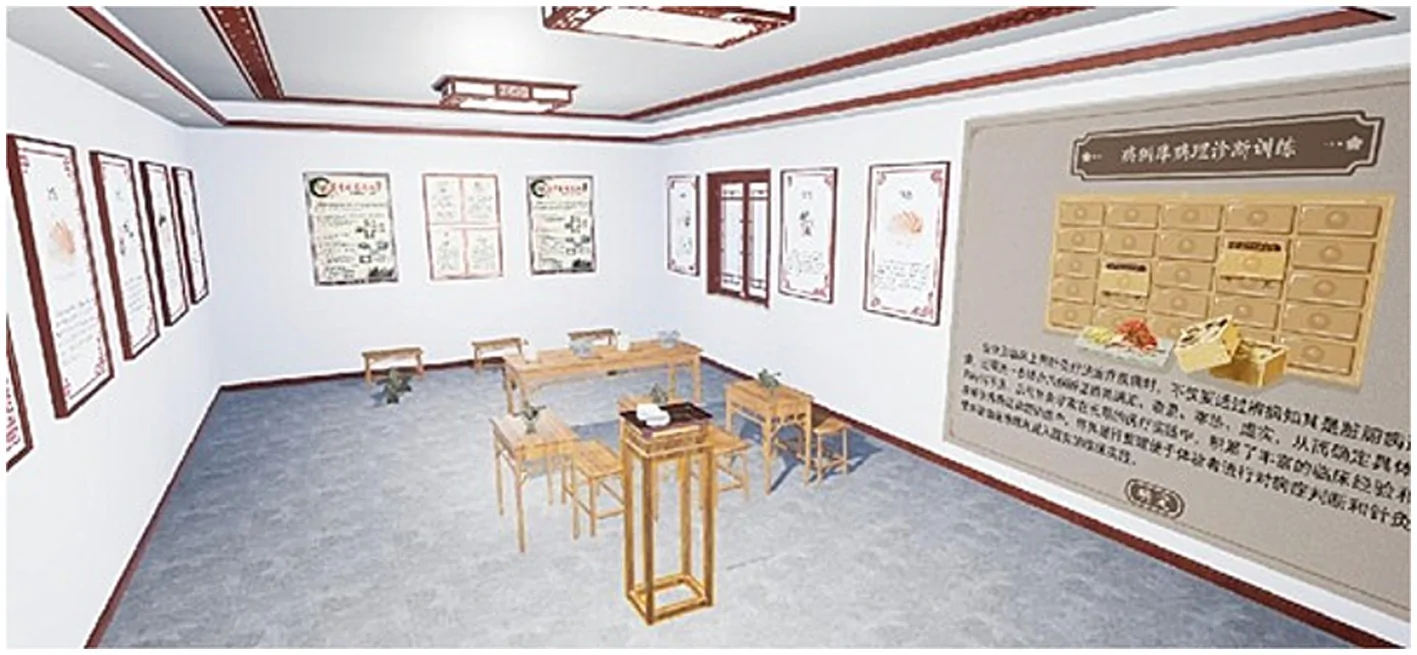
Example of a diagnostic scenario presented in the PainEaseVR case bank.

**Figure 9:**
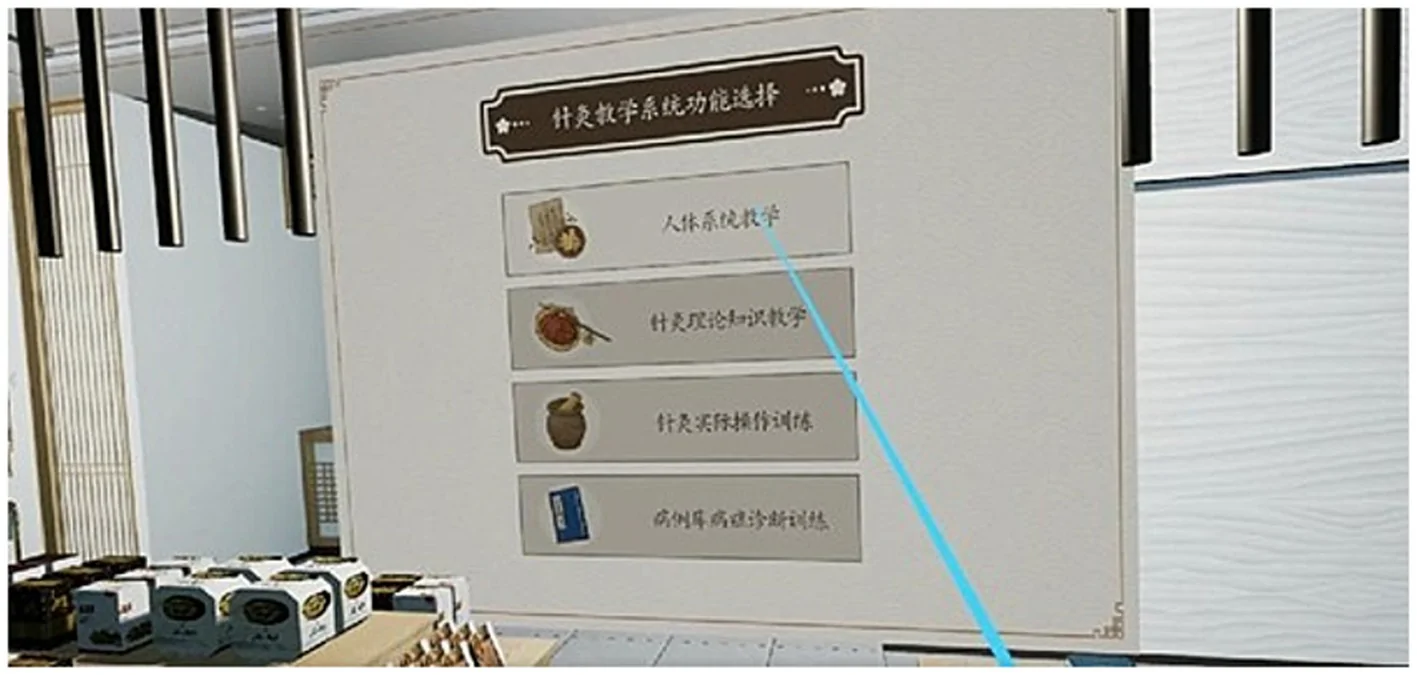
Navigation user interface of the PainEaseVR system.

**Figure 10:**
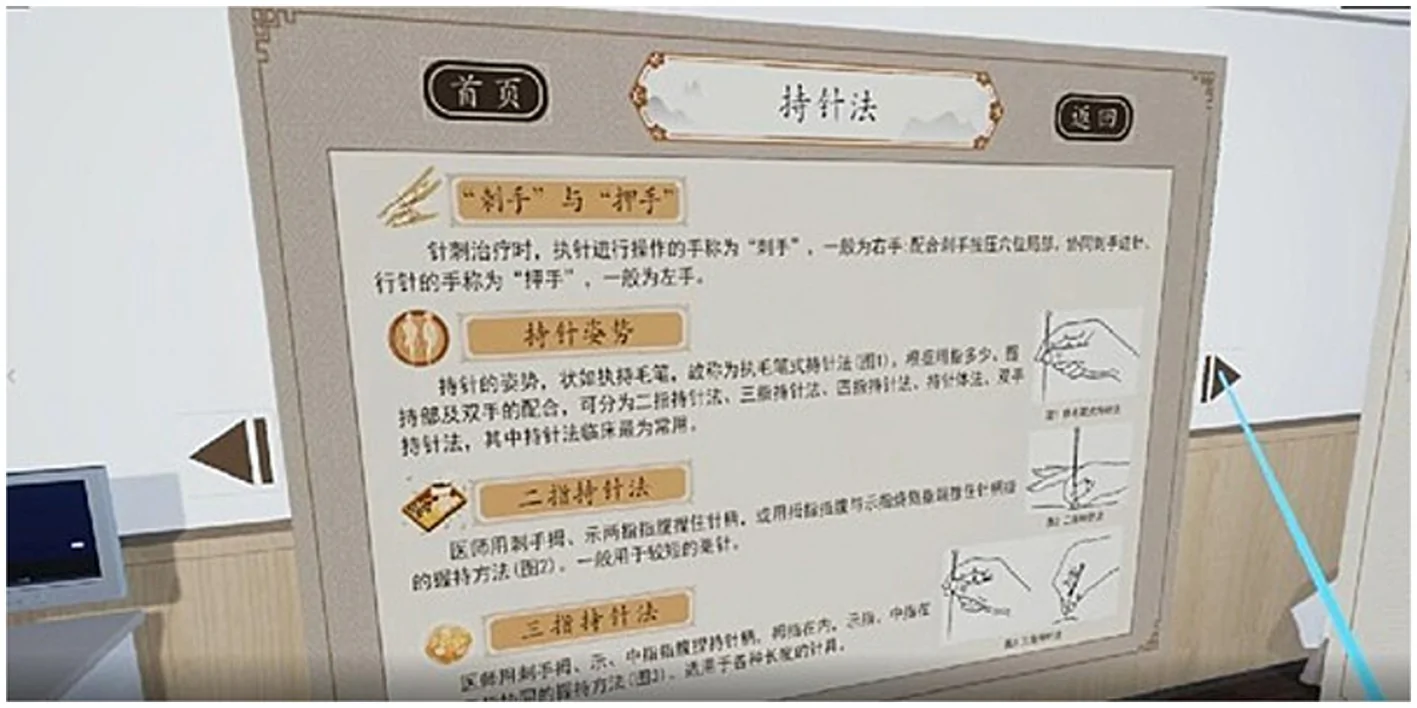
Needle-operation learning interface in the PainEaseVR simulation module.

The case bank diagnostic training scenario is designed to replicate the genuine pressure and atmosphere of an examination environment, inspired by traditional acupuncture classroom layouts (see Figure 7). Representative views of the module, including a diagnostic scenario and related user interfaces, are presented in Figures 8–10. This setup immerses users in a realistic context that reflects actual diagnosis and treatment situations, enhancing their readiness and confidence. This scenario functions as a comprehensive acupuncture evaluation system, allowing users to apply the theoretical knowledge gained from previous educational modules. These lessons cover a wide range of acupuncture principles and practices, providing a solid foundation in the subject. The scenario includes extensive simulation exercises that illustrate various diseases, providing users with a practical setting to apply their knowledge. Participants are encouraged to use their understanding of symptoms and associated treatment plans through interactive assessments of their learning outcomes. This interactive component is crucial, enabling users to engage actively in the diagnostic process and make informed decisions based on their understanding of acupuncture techniques. The scenario promotes the development of critical thinking and problem-solving skills by simulating real diagnostic challenges, which are essential for effective acupuncture practice.

## 4 Methodology

### 4.1 Study design and participants

A stratified, randomized, posttest-only, comparative design was used. Sixty third-year undergraduate students majoring in Traditional Chinese Medicine participated in the study. All had previously completed foundational acupuncture coursework. Stratification was conducted using two pre-allocation indicators available in the course context: (1) prior academic performance in acupuncture-related coursework and (2) instructor-rated foundational practical competence from prior laboratory sessions. These indicators were used only to balance the groups at allocation. After stratification, students were randomly assigned to either the VR group (*n* = 30) or the traditional group (*n* = 30). This random allocation, coupled with stratification, ensured that both groups began the trial with comparable levels of expertise, allowing for a fair evaluation of the effectiveness of the two different training methods. A structured description of the educational intervention, reported in accordance with the GREET checklist (32), is provided in Table 1.

**Table 1:** GREET checklist for the educational intervention.

No.	GREET item	Information reported for the present study
1	Intervention	This study evaluated a two-week educational intervention comparing virtual reality (VR)-based acupuncture training with traditional acupuncture instruction among 60 third-year undergraduate students majoring in Traditional Chinese Medicine. Both groups completed the same formal curriculum, consisting of 12 h of instruction: 6 h of theoretical teaching and 6 h of practical training.
2	Theory	The VR intervention was informed by a constructivist learning approach and was designed to promote active learning through immersive interaction. Students engaged with three-dimensional anatomical models, explored meridians and acupoints, completed simulated clinical scenarios, made procedural decisions, and received immediate feedback within the virtual environment.
3	Learning objectives	The intervention aimed to develop theoretical knowledge, practical acupuncture skills, procedural competence, and learner confidence. Content included meridian and acupoint distribution, standardized acupuncture procedures, indications and contraindications, safety and ethics, acupoint localization, needle insertion and manipulation, hygiene, sharps disposal, and complete simulated clinical procedures.
4	EBP content	Not applicable. The intervention did not explicitly teach the five steps of evidence-based practice—ask, acquire, appraise, apply, and assess. Instead, it focused on acupuncture theory and procedural-skills training.
5	Materials	The traditional group used lectures, multimedia courseware, schematic diagrams from the textbook *Acupuncture and Moxibustion*, anatomical models, silicone training arms, and peer practice. The VR group used a headset, controllers, and a custom internal VR platform (PainEaseVR; no formal version number), together with custom male and female three-dimensional body models, manually annotated acupoints, virtual needles, simulated cases, automated scoring, and real-time feedback.
6	Educational strategies	Traditional instruction included lectures, textbook-based visual materials, instructor demonstrations, case discussions, peer practice, supervised group practice, and verbal feedback. VR instruction included interactive three-dimensional exploration, scenario-based learning, simulated procedural practice, automated scoring, real-time feedback, reflection, and complete clinical simulations.
7	Incentives	Participants received a supermarket coupon valued at RMB 20 as compensation for participation.
8	Instructors	The traditional intervention was delivered by acupuncture instructors through face-to-face teaching. In the VR group, no instructor delivered the formal instructional content directly; instead, the learning content was delivered through the VR platform as self-directed training. A researcher was present during the VR sessions to monitor intervention delivery, provide technical assistance, and respond to participant discomfort.
9	Delivery	The traditional intervention was delivered face-to-face in instructor-led sessions that included lectures, demonstrations, guided discussions, supervised practice, and verbal feedback. The VR intervention was delivered as self-directed individual learning through the VR system and included interactive three-dimensional learning, simulated cases, procedural exercises, and automated real-time feedback.
10	Environment	The theoretical component of the traditional intervention was delivered in a university classroom equipped with computers, multimedia facilities, and a projector. Practical acupuncture-skills training was conducted in a clinical skills laboratory equipped with acupuncture beds, anatomical models, silicone training arms, acupuncture needles, and disinfection materials. Students practiced under instructor supervision and received demonstrations and verbal feedback. The VR intervention was conducted in a controlled teaching environment using VR equipment and simulation technology. A researcher remained available during the sessions to monitor delivery, address technical issues, and respond to participant discomfort.
11	Schedule	Both groups completed 12 h of formal instruction over 2 weeks, consisting of 6 h of theory and 6 h of practical training. The theoretical modules included meridians and acupoint distribution (approximately 1.5 h), standardized acupuncture operation (approximately 2 h), indications and contraindications (approximately 1.5 h), and safety, ethics, and complications (approximately 1 h). The practical modules included acupoint localization (1.5 h), needle manipulation and insertion (2 h), pre- and post-acupuncture procedures (1 h), and simulated clinical operation (1.5 h).
12	Instructor contact and self-directed time	Students in the traditional group completed 12 h of instructor-led learning, comprising 6 h of theoretical teaching and 6 h of practical instruction. Students in the VR group completed the same 12-h formal curriculum as self-directed VR learning. In addition, the VR group received an approximately 1-h standardized orientation to the equipment, controllers, and navigation functions before formal training began.
13	Planned changes or adaptations	All participants in the VR group received the same standardized orientation before formal training and were permitted to stop temporarily if they experienced discomfort. These procedures were prespecified components of the VR protocol rather than individualized adaptations. No participant-specific modifications were planned.
14	Unplanned changes	No unplanned modifications to the intervention were reported during the study. The intervention content, delivery modes, and overall schedule remained unchanged throughout the trial.
15	Attendance	Attendance was monitored during the intervention period. All randomized participants completed the study procedures as planned.
16	Delivery fidelity	Intervention delivery was monitored through researcher oversight during the sessions to ensure that the planned content and duration were implemented as scheduled. In the traditional group, this involved confirming delivery of the predefined theoretical and practical modules. In the VR group, this involved monitoring completion of the corresponding self-directed modules and access to the programmed automated feedback.
17	Schedule fidelity	Both groups completed the planned 12-h intervention over 2 weeks, including 6 h of theoretical instruction and 6 h of practical training. The scheduled structure and intended duration were maintained throughout the study.

The study was conducted in a controlled teaching environment. Both groups received the same overall instructional duration over 2 weeks (12 h total, 6 h theory and 6 h practical training), but the modes of delivery differed. The study was conducted in accordance with institutional ethical requirements, and all participants provided informed consent prior to participation. The study aimed to compare the two instructional approaches—traditional and VR—in two main components: theoretical instruction and practical training. By evaluating how each method was associated with the acquisition of theoretical knowledge and practical skills, the research sought to provide insights into their potential roles in developing confidence and competence in acupuncture techniques. Before the formal VR sessions, participants in the VR group received an approximately one-hour standardized orientation to the headset, controllers, and navigation functions. This introductory session was intended to reduce technology-related confusion and ensure that participants could operate the system before training began. The orientation focused on equipment operation rather than acupuncture content and was not included in the 12-h formal intervention. No equivalent orientation session was provided to the traditional group. Participants were allowed to stop temporarily if they experienced discomfort. No severe cybersickness events were recorded during the sessions.

### 4.2 Theory teaching—traditional vs. VR

The theory teaching component of the study consisted of 6 h for both the control group and the experimental group, structured into four detailed modules with specific timings: meridians and acupoints distribution (approximately 1.5 h), standardized acupuncture operation (approximately 2 h), indications and contraindications (approximately 1.5 h), and safety, ethics, and complications (approximately 1 h). In the meridians and acupoints distribution module, the traditional method involved lectures supplemented by multimedia courseware and schematic diagrams from the textbook “Acupuncture and Moxibustion,” enabling students to visualize the pathways and locations of various acupoints. In contrast, the VR method enabled participants to explore a 3D virtual environment where they could interactively visualize and manipulate meridians and acupoints. For the standardized acupuncture operation module, the traditional group received demonstrations from instructors on the complete acupuncture process, including disinfection protocols and the order of procedures, which were reinforced with multimedia illustrations. Conversely, the VR group experienced simulated acupuncture procedures in a virtual environment and received real-time feedback on their techniques. In the indications and contraindications module, the traditional approach involved case discussions that emphasized the rational selection of acupuncture points and risk prevention. The VR method, however, used scenario-based learning, in which participants encountered virtual case studies requiring them to make decisions about acupuncture points based on specific patient profiles, thereby bridging theory and practice. Finally, the safety, ethics, and complications module in the traditional method emphasized effective patient communication and established safety standards through lectures and guided discussions. In the VR setting, this module facilitated immersive discussions on ethical dilemmas and case analyses, encouraging active participation and reflection on patient care responsibilities.

### 4.3 Practical training—traditional vs. VR

The practical training component also consisted of 6 h, with consistent content across both groups but varying teaching methods. It was divided into four key modules: acupoint localization (1.5 h), needle manipulation and insertion (2 h), pre- and post-acupuncture operations (1 h), and simulated clinical operation (1.5 h). In the acupoint localization module, the traditional method involved students practicing palpation and precise localization of commonly used acupoints on each other and anatomical models. Instructors guided the students, providing demonstrations and immediate feedback to ensure proper technique. In contrast, the VR group engaged with a VR simulation platform featuring 3D visualizations of acupoints. Participants could interact with the virtual environment to practice localization and receive real-time feedback on their performance. The needle manipulation and needle insertion module focused on training students in the angle, depth, and technique of needle insertion, as well as manipulation and withdrawal skills. The control group used a silicone training arm, during which instructors demonstrated proper techniques, followed by group practice with real-time verbal feedback. Conversely, the VR group practiced needle insertion techniques using the simulation platform, which provided automated scoring and real-time interactive feedback. This immersive experience allowed participants to experiment with different techniques in a risk-free environment. During the one-hour pre- and post-acupuncture module, participants learned essential procedures for hand hygiene, skin disinfection, and proper sharps disposal. The traditional group engaged in practical demonstrations and instructor-led discussions, emphasizing the importance of hygiene and safety protocols. The VR group practiced these procedures in a simulated environment, allowing them to visualize and execute each step while receiving feedback on their performance. Finally, the simulated clinical operation module required participants to complete the entire acupuncture process, from preparation to execution and aftercare. The VR group underwent a fully immersive simulation that mimicked real clinical scenarios, providing them with comprehensive feedback on their performance and areas for improvement.

### 4.4 Evaluation of teaching methods

The evaluation of learning consisted of two primary components: a theoretical knowledge test and a practical skills assessment, both conducted immediately following the respective teaching methods. Participants from both groups completed a theoretical knowledge test consisting of 12 multiple-choice questions (Supplementary Appendix I), each worth 1 point for a correct answer, designed to assess their understanding of key acupuncture concepts. The questions covered essential topics, including meridian theory, acupoint selection, operational norms, and safety knowledge. To ensure the quality and relevance of the assessment, three senior experts in acupuncture education reviewed the questions, and the content validity index (I-CVI) was calculated. A high I-CVI score of 0.83 or greater indicated that the test items were valid and effectively measured the intended knowledge areas (33).

Additionally, participants underwent a practical skills assessment, conducted individually in the experimental room. This assessment used an 18-item practical skills checklist (Supplementary Appendix II) to evaluate key practical skills, including hand hygiene, accuracy of acupoint positioning, needle insertion techniques, and operative records. Each skill was scored using a three-point scale: 0 for incomplete, 1 for partially correct, and 2 for entirely accurate, allowing for a maximum total score of 36. For the practical assessment, participants in both groups used a silicone training arm to apply their practical skills. Each student was required to independently complete a standardized simulation task within a 6–8-min time limit. Two licensed acupuncture assessors independently evaluated participants’ practical performance using a predefined 18-item checklist. Both assessors were blinded to group allocation. All assessments were conducted in a standardized environment, without access to VR equipment or instructional materials used during training. Participants were identified solely by study codes and were instructed not to disclose their training method. Assessors completed their ratings independently and did not confer regarding scores prior to submission. Inter-rater consistency was examined using the Intraclass Correlation Coefficient (ICC). The ICC value was above 0.75, indicating good agreement between raters and supporting the reliability and reproducibility of the scoring process (34).

### 4.5 Students’ feedback

After completing the assessments, students were invited to fill out a questionnaire to provide their subjective evaluation of the teaching methods. The instructor created an 11-question satisfaction survey designed to collect feedback on various aspects of the instructional approaches. This survey was designed to gage students’ perceptions of the program, including their understanding of the material, levels of enjoyment and motivation, and the relevance and applicability of the knowledge and skills they acquired for their future careers. Students rated their perceptions of teaching quality on a 5-point Likert scale, from 1 (strongly disagree) to 5 (strongly agree). This allowed them to express their agreement or disagreement with various statements regarding the teaching methods.

### 4.6 Data analysis

The data were entered and analyzed using SPSS version 20.0. PainEaseVR is a custom internal tool and has no formal version number. Internal consistency of the study measures was assessed using Cronbach’s alpha. The 18-item practical skills assessment demonstrated good internal consistency, with Cronbach’s alpha coefficients of 0.839 in the experimental group and 0.890 in the control group. For the learning experience survey, Cronbach’s alpha was 0.792 for the control group and 0.803 for the experimental group. The theoretical knowledge test consisted of multiple-choice items scored as correct or incorrect; therefore, Cronbach’s alpha is equivalent to KR-20 for this measure. The corresponding coefficients were 0.619 for the control group and 0.625 for the experimental group, indicating modest internal consistency. Although this value was below the conventional 0.70 criterion, coefficients around 0.60 may be considered provisionally acceptable in exploratory research and for relatively short, broad-content knowledge tests (35). Descriptive statistics, including means and standard deviations (SD), were used to summarize the data. Independent-samples t-tests were conducted to compare differences between the two teaching methods on theoretical knowledge, practical skills, and student feedback outcomes. For the 11 feedback items, a Bonferroni correction was applied, resulting in an adjusted significance threshold of *α* = 0.0045. For the remaining analyses, statistical significance was set at *p* < 0.05. Cohen’s d and 95% confidence intervals were calculated to indicate the magnitude and precision of between-group differences.

## 5 Results

The evaluation scores revealed significant differences between the two teaching methods (see Table 2). In the theoretical knowledge assessment, students in the experimental group achieved a mean score of 10.08 (SD = 0.99), whereas the control group obtained a mean score of 9.04 (SD = 1.31). This between-group difference was statistically significant (*t*(58) = 3.484, *p* < 0.001) and corresponded to a large effect size (Cohen’s *d* = 0.90, 95% CI [0.36, 1.43]), indicating that the VR-based instructional method was associated with higher theoretical knowledge scores. Similarly, the practical skills assessment showed a marked difference between groups. The experimental group attained a mean score of 32.52 (SD = 2.28), compared with 28.62 (SD = 2.77) in the control group. This difference was also statistically significant (*t*(58) = 5.947, *p* < 0.001) and was associated with a large effect size (Cohen’s *d* = 0.90, 95% CI [0.36, 1.43]). Overall, students in the VR group outperformed those in the traditional group on both theoretical knowledge and practical skills assessments, indicating that the VR-based instructional approach was associated with higher performance than the traditional instructional approach in this study.

**Table 2:** Comparison of evaluation scores between teaching methods assessments.

Evaluation	Group	M	SD	*t*	df	*p*	Cohen’s *d* (95% CI)
Theoretical knowledge	Experimental	10.08	0.99	3.484	58	<0.001	0.90 [0.36, 1.43]
	Control	9.04	1.31				
Practical skills	Experimental	32.52	2.28	5.947	58	<0.001	0.90 [0.36, 1.43]
	Control	28.62	2.77				

The feedback survey also showed that students in the experimental group reported more positive learning experiences than those in the control group across all 11 items (see Table 2). All between-group comparisons were statistically significant at *p* < 0.001. To control for multiple comparisons, a Bonferroni correction was applied across the 11 feedback items. All differences remained statistically significant after this correction. Overall, the experimental group reported higher ratings of focus, concentration, self-paced learning, timely feedback, active participation, understanding of acupuncture procedures, ability to apply learning to practice, satisfaction, engagement, and perceived learning benefit. For example, the experimental group reported higher levels of focus during the learning session (M = 4.50 vs. 3.30), greater active participation (M = 4.57 vs. 3.33), and higher overall satisfaction with the learning experience (M = 4.60 vs. 3.47). These findings indicate that the VR-based instructional approach was associated not only with higher performance in theoretical knowledge and practical skills, but also with more positive student perceptions of learning effectiveness.

## 6 Discussion

The purpose of this study was to evaluate the association of VR training with the theoretical understanding and practical competencies of acupuncture students. To achieve this, a comparative method was employed to contrast traditional teaching methods with VR-based instruction. Two hypotheses were formulated to determine whether VR-enhanced teaching would be associated with higher theoretical understanding and practical skills than traditional methods. The findings indicate that integrating VR was associated with higher immediate posttest scores in both theoretical knowledge and practical skills in acupuncture education. The experimental group, which received VR training, outperformed the control group in both assessments, achieving higher mean scores. These results align with a growing body of literature indicating that immersive technologies facilitate deeper learning and skill acquisition. For instance, studies have shown that VR can create engaging learning experiences that motivate students and enhance their comprehension of complex concepts (36–40).

These findings are particularly relevant when examined through the lens of the CTML. According to CTML, effective learning occurs when information is presented through a combination of visual and auditory modalities, enabling students to process information more deeply (41). In our study, the VR environment offered a rich, interactive context that merged visual and tactile experiences, thereby enhancing cognitive processing and retention of acupuncture concepts. This supports prior research highlighting the benefits of multimedia learning environments for improving educational outcomes (42–46).

Furthermore, the results can also be interpreted through Flow Theory, which posits that optimal learning experiences occur when individuals are fully immersed and engaged in a task that balances challenge and skill level (47). The VR training likely fostered an environment conducive to achieving this flow state, as students reported higher levels of focus and satisfaction. The immediate feedback provided in VR scenarios further enhanced this flow experience, allowing students to adjust their techniques in real time—an essential factor for mastering practical skills. Compared with traditional methods, which may lack the immersive and interactive elements of VR, our findings suggest that VR may offer educational advantages in traditional Chinese medicine training, particularly in acupuncture. Traditional teaching often relies on passive learning techniques that may not engage students as effectively or provide the immediate feedback necessary for skill development. As demonstrated in our study, VR training not only improved theoretical knowledge but also translated into practical competencies, underscoring its potential as a transformative tool in acupuncture education.

Furthermore, feedback from students in the experimental group indicated markedly higher satisfaction across various aspects, including engagement, comprehension, pace, and self-confidence (48). The increased mean scores indicate that students perceived themselves as more focused and better equipped to regulate their learning rate, which is consistent with constructivist perspectives and multimedia learning principles (49). The capacity of VR to integrate multimodal inputs with learner-directed navigation promotes active knowledge creation (50). By integrating visual and auditory information, VR facilitates cognitive processing and enables learners to form meaningful connections, ultimately enhancing retention and comprehension (51). Additionally, the elevated ratings for prompt response and overall contentment underscore the distinctive benefits of VR in fostering an interactive and responsive teaching setting (52). This conclusion is corroborated by current literature, which highlights the capacity of immersive technology to enhance academic performance by empowering learners and augmenting their sense of agency (53). The experimental group’s increased confidence in understanding and performing acupuncture techniques highlights VR’s ability to connect theoretical learning with practical application, providing safe practice opportunities that are often unavailable in conventional environments (46). The integration of cognitive, emotional, and physical advantages establishes VR as a potentially valuable instrument for experiential learning in procedural fields (54). These findings indicate that VR-based learning environments can substantially enhance the educational experience by fostering engagement, aiding skill acquisition, and facilitating the application of knowledge in real-world contexts (55).

## 7 Implications and limitations

The findings of this study have significant implications both theoretically and practically. The enhanced theoretical knowledge and practical skills demonstrated by students trained using VR suggest that immersive learning environments were associated with higher scores compared to traditional pedagogical approaches. From a theoretical perspective, the results challenge conventional notions of learning in medical education, emphasizing the need to integrate technology-driven methods that enhance student engagement and understanding. This aligns with contemporary educational theories that advocate experiential and interactive learning, suggesting that incorporating VR can facilitate deeper cognitive processing and retention of complex concepts, such as meridian theory and acupoint localization. In practice, the VR group’s superior performance in both theoretical assessments and practical skills suggests that educational institutions should consider adopting VR technology as a supplementary component of their training programs. The immersive nature of VR not only allows students to visualize and manipulate anatomical structures and procedures but also provides a safe environment for practice without the risks associated with real-life clinical scenarios. This has profound implications for skill acquisition, as students can experiment and receive immediate feedback, which is crucial for mastering techniques such as needle insertion and patient interaction. Furthermore, the positive student feedback regarding engagement and satisfaction suggests that VR can enhance the overall learning experience, fostering a more motivated and confident cohort of future practitioners. As acupuncture education evolves, integrating VR technology may not only improve educational outcomes but also prepare students for the demands of modern healthcare, where technological proficiency is increasingly essential (Table 3).

**Table 3:** Comparison of student feedback ratings between the control and experimental group.

Survey item	Control group M (SD)	Experimental group M (SD)	Mean difference (Experimental – Control) [95% CI]	*t*(58)	Adjusted significance	Cohen’s *d* [95% CI]
I was able to stay focused during the learning session	3.30 (0.47)	4.50 (0.51)	1.20 [0.95, 1.45]	9.48	<0.001	2.45 [1.77, 3.12]
The learning environment helped me concentrate on the practice	3.50 (0.51)	4.43 (0.50)	0.93 [0.67, 1.19]	7.13	<0.001	1.84 [1.23, 2.45]
I was able to follow the learning process at my own pace	3.67 (0.48)	4.43 (0.50)	0.76 [0.51, 1.01]	6.01	<0.001	1.55 [0.97, 2.13]
I received timely feedback during practice or instruction	3.50 (0.51)	4.37 (0.49)	0.87 [0.61, 1.13]	6.74	<0.001	1.74 [1.14, 2.34]
I was able to participate in the learning session actively	3.33 (0.48)	4.57 (0.50)	1.24 [0.99, 1.49]	9.80	<0.001	2.53 [1.85, 3.21]
I well understood the content and structure of acupuncture procedures	3.47 (0.51)	4.57 (0.50)	1.10 [0.84, 1.36]	8.44	<0.001	2.18 [1.54, 2.82]
I could apply effectively what I learned to real practice	3.43 (0.50)	4.47 (0.51)	1.04 [0.78, 1.30]	7.98	<0.001	2.06 [1.43, 2.69]
I well understood the key steps of the acupuncture operation	3.57 (0.50)	4.60 (0.50)	1.03 [0.77, 1.29]	7.98	<0.001	2.06 [1.43, 2.69]
I am satisfied with the overall learning experience	3.47 (0.51)	4.60 (0.50)	1.13 [0.87, 1.39]	8.67	<0.001	2.24 [1.59, 2.89]
The method used kept me engaged throughout the session	3.63 (0.49)	4.53 (0.51)	0.90 [0.64, 1.16]	6.97	<0.001	1.80 [1.20, 2.40]
This approach helped me learn better than I expected	3.53 (0.51)	4.50 (0.51)	0.97 [0.71, 1.23]	7.37	<0.001	1.90 [1.29, 2.52]

Values are presented as mean (SD). Group comparisons were conducted using independent-samples *t*-tests. To control for multiple comparisons across the 11 feedback items, a Bonferroni correction was applied. All between-group differences remained statistically significant after Bonferroni correction. Cohen’s d is reported as the standardized mean difference, with 95% confidence intervals.

Several limitations should be acknowledged. First, participants were recruited exclusively from a single institution, and the sample size was modest. No formal *a priori* power analysis was conducted, and the sample size was determined by the number of eligible students available during the study period. Therefore, the study may have had limited power to detect smaller effects, and the effect-size estimates may be imprecise. Future multicenter studies should use an a priori power analysis based on a prespecified primary outcome. Second, the posttest-only design precluded direct assessment of individual changes from baseline. Although stratified randomization balanced prior academic performance and instructor-rated practical competence, unmeasured baseline differences between groups may persist. Third, the intervention duration was limited to 2 weeks, and outcomes were assessed immediately following training. The study did not evaluate whether observed differences persisted over time or translated to performance with actual patients. While the VR system simulated acupoint localization and acupuncture procedures, it could not fully replicate tactile sensations, tissue resistance, patient communication, or the complexity of clinical practice. Therefore, superior performance in the virtual environment should not be interpreted as evidence of equivalent clinical competence. Fourth, this study compared two comprehensive instructional approaches rather than isolating the effect of VR technology alone. In addition to modality, the groups differed in instructional structure and feedback source. The VR group participated in self-directed learning with real-time programmed feedback and automated scoring, whereas the traditional group received instructor-led teaching, demonstrations, supervision, and verbal feedback. Consequently, the observed differences likely reflect the combined influence of immersive technology, self-directed practice, feedback frequency and format, and instructor involvement. Both groups completed an identical 12-h formal acupuncture curriculum; however, the VR group also participated in an approximately one-hour equipment orientation session that was not provided to the traditional group. Although the orientation did not include acupuncture content, it increased the VR group’s overall study exposure and may have reduced technology-related uncertainty or enhanced familiarity with the training environment. Future research should employ attention- and time-matched control conditions and, where feasible, standardize feedback frequency and instructional contact to more effectively isolate the specific effects of VR. Next, the theoretical test encompassed several acupuncture knowledge domains but included a relatively small number of items. Because it was designed as a multidomain, criterion-referenced assessment rather than a unidimensional scale, internal-consistency estimates should be interpreted with caution. Although experts evaluated content validity and practical performance was independently rated, further research is needed to examine the psychometric properties of these assessments in larger samples. Future multicenter studies should incorporate baseline assessments, longer-term follow-up, objective clinical performance outcomes, and comparisons that more effectively isolate the effects of VR technology from instructional delivery. Finally, participants’ responses and performance may have been influenced by the novelty of VR. As this study measured outcomes immediately following a brief intervention, it is uncertain whether the observed benefits would persist as participants gain familiarity with the technology. Future research should incorporate delayed retention assessments, such as evaluations conducted several weeks or months after training, as well as transfer tests in a standardized non-VR clinical simulation. If the initial advantage is primarily attributable to novelty, it is expected to diminish in delayed or non-VR assessments. Persistent differences would suggest more durable learning. Future studies should systematically measure engagement, motivation, perceived novelty, and cybersickness across multiple sessions to determine whether these responses decline as participants become more familiar with the system. Including an active control group using an alternative digital learning technology, matched for equipment exposure, instructional time, feedback frequency, and researcher attention, would facilitate differentiation between the effects of immersion and general technological novelty. Employing a counterbalanced crossover design would enable participants to experience both instructional approaches. Because prior acupuncture knowledge and skills cannot be eliminated through a standard washout period, each phase should employ distinct but equivalent learning modules and explicitly assess sequence, period, and carryover effects.

## 8 Conclusion

In conclusion, this study aimed to evaluate the association of VR with instructional outcomes in acupuncture education, guided by two primary hypotheses. The findings were consistent with both hypotheses at the level of immediate posttest group differences, indicating that students trained with VR achieved significantly higher immediate posttest scores in theoretical knowledge of acupuncture and practical skills than students receiving traditional instruction. This research demonstrates that VR was associated with higher scores as a supplementary instructional approach in acupuncture education. Additionally, students who engaged in VR training reported greater engagement, satisfaction, and confidence in their understanding and application of acupuncture techniques compared to their peers in traditional learning environments. This suggests that incorporating VR into educational frameworks can provide a more immersive and interactive learning experience, with the potential to contribute to better preparation of future practitioners in Traditional Chinese Medicine.

## Data Availability

The raw data supporting the conclusions of this article will be made available by the authors, without undue reservation.
