# Supplementary material for: Immersive virtual reality for acupuncture knowledge and practical skills: a comparative educational study
Source: Front Public Health. 2026 Aug 27;14:1922688. doi: 10.3389/fpubh.2026.1922688 (PMC13558152; doi:10.3389/fpubh.2026.1922688)
Supplement: Supplementary file 1 [file Table_1.DOCX]

**Appendix I. Theoretical Knowledge Assessment Questions**

Scoring: 1 point for the single best answer; 0 for an incorrect, blank, or multiple response; total 0–12.

| **No.** | **Question and options** | **Key** | **Domain** |
| --- | --- | --- | --- |
| 1 | Which is a common indication for Yingxiang (LI20)? A. Red and painful eyes B. Nasal obstruction or sinus symptoms C. Tinnitus or hearing loss D. Soreness of the lower back and knees | B | Point indication |
| 2 | Which statement about the Stomach Meridian of Foot-Yangming is correct? A. It begins at the lateral side of the second toe B. It terminates at Renying (ST9) C. It runs only through the upper limb D. It descends to Lidui (ST45) at the lateral side of the second toe | D | Meridian course |
| 3 | What is the standard location of Quchi (LI11)? A. With the elbow flexed, at the midpoint of the line connecting Chize (LU5) and the lateral epicondyle of the humerus B. At the medial end of the cubital crease C. At the midpoint of the dorsal wrist crease D. Posteroinferior to the medial epicondyle of the humerus | A | Point location |
| 4 | Under routine acupuncture safety teaching, in which situation should strong stimulation at Hegu (LI4) be avoided? A. Mild nasal obstruction B. Pregnancy, especially before term C. Shoulder stiffness D. A common cold in a non-pregnant person | B | Safety |
| 5 | The traditional combination of Hegu (LI4) and Taichong (LR3) is known as: A. Front-Mu and Back-Shu pairing B. Yuan-Source and Luo-Connecting pairing C. Four Gates D. Eight Confluent point pairing | C | Point combination |
| 6 | Insertion with the needle approximately perpendicular (90 degrees) to the skin is called: A. Perpendicular insertion B. Oblique insertion C. Transverse insertion D. Lifting-thrusting | A | Insertion angle |
| 7 | What is the basic movement in lifting-thrusting manipulation? A. Repeatedly lifting and advancing the needle along the direction of insertion B. Rotating the handle without changing needle position C. Pressing only the skin around the insertion site D. Changing the patient's position without moving the needle | A | Manipulation |
| 8 | When a needle is retained by local muscle tension, the first response should be to: A. Withdraw it forcefully B. Ask the patient to relax, wait briefly, and withdraw slowly after the muscle relaxes C. Insert the needle more deeply D. Bend the needle before withdrawal | B | Adverse-event response |
| 9 | Which point is not on the first posterior line of the Bladder Meridian of Foot-Taiyang? A. Dazhu (BL11) B. Fengmen (BL12) C. Feishu (BL13) D. Gaohuang (BL43) | D | Point distribution |
| 10 | What direction is commonly taught for needling Shuigou (GV26)? A. Perpendicularly downward B. Obliquely upward C. Obliquely downward D. Transversely toward the ear | B | Needling direction |
| 11 | Which is a common predisposing factor for acupuncture-associated fainting? A. Being relaxed after an adequate meal B. Excessive hunger or fatigue C. Using an appropriate supine position D. Using qualified sterile single-use needles | B | Fainting prevention |
| 12 | What is the standard location of Zusanli (ST36)? A. On the anterolateral leg, 3 cun below Dubi (ST35), on the line connecting ST35 and Jiexi (ST41) B. On the medial leg, 3 cun below Yinlingquan (SP9) C. At the midpoint of the popliteal crease D. Three cun above the lateral malleolus, anterior to the fibula | A | Point location |

**Appendix II. Practical Skills Assessment Checklist**

Scoring: 2 = complete, correct, and standardized; 1 = partially correct with a correctable deviation and no critical safety error; 0 = not completed, incorrect, or a critical safety error. Total 0–36.

| **Code** | **Domain** | **Item** | **Full-score criterion** | **Score** |
| --- | --- | --- | --- | --- |
| A1 | Preparation | Hand hygiene | Performs hand hygiene before contact with the participant and sterile equipment. | 0–2 |
| A2 | Preparation | Equipment check | Checks needle size, sterile single-use packaging and expiry; prepares sterile dry cotton and a sharps container. | 0–2 |
| A3 | Preparation | Communication and position | Confirms the participant, explains the procedure, obtains cooperation, and selects a stable and safe position. | 0–2 |
| A4 | Preparation | Skin antisepsis | Disinfects from the intended insertion site according to protocol, allows drying, and avoids recontamination. | 0–2 |
| B1 | Point location | Locating method | Uses surface anatomy and proportional bone-cun measurement appropriately. | 0–2 |
| B2 | Point location | Accuracy | Locates the assigned point within the standard location and the prespecified assessment tolerance. | 0–2 |
| B3 | Point location | Verification | Rechecks point name, side and surface location before insertion; no point or side error occurs. | 0–2 |
| C1 | Needling | Needle handling and asepsis | Holds the needle steadily without contaminating the shaft or other sterile parts. | 0–2 |
| C2 | Needling | Insertion angle | Selects perpendicular, oblique or transverse insertion as required for the assigned point and scenario. | 0–2 |
| C3 | Needling | Insertion depth | Uses a safe depth appropriate to the assigned point, body type and simulated scenario; no excessive depth. | 0–2 |
| C4 | Needling | Manipulation | Performs the required lifting-thrusting or rotation with appropriate direction, amplitude and frequency. | 0–2 |
| C5 | Needling | Retention and needle count | Maintains stable needles during retention and confirms a matching needle count before and after the procedure. | 0–2 |
| C6 | Needling | Adverse-event response | Correctly identifies and states or simulates safe responses to fainting, stuck needle and bent needle. | 0–2 |
| C7 | Needling | Monitoring and communication | Asks about symptoms and observes pallor, sweating or dizziness; stops and responds promptly if abnormal signs occur. | 0–2 |
| D1 | Removal and aftercare | Needle removal | Withdraws smoothly, applies sterile dry cotton immediately, and reconfirms the needle count. | 0–2 |
| D2 | Removal and aftercare | Site care | Applies appropriate pressure until bleeding or oozing stops and inspects the site. | 0–2 |
| D3 | Removal and aftercare | Waste disposal | Places used needles directly in a sharps container without recapping or hand sorting; segregates other waste. | 0–2 |
| D4 | Removal and aftercare | Documentation | Records points, side, main procedures, adverse events and their management. | 0–2 |
